# Long-chain vitamin K2 production in *Lactococcus lactis* is influenced by temperature, carbon source, aeration and mode of energy metabolism

**DOI:** 10.1186/s12934-019-1179-9

**Published:** 2019-08-06

**Authors:** Yue Liu, Eric O. van Bennekom, Yu Zhang, Tjakko Abee, Eddy J. Smid

**Affiliations:** 1 Food Microbiology, Wageningen University and Research, PO Box 17, 6700 AA Wageningen, The Netherlands; 2 BU Veterinary Drugs, RIKILT, Wageningen University and Research, Akkermaalsbos 2, 6708 WB Wageningen, The Netherlands; 3 Present Address: Shanghai, People’s Republic of China

**Keywords:** Menaquinone, Long-chain MKs, Vitamin K2, Natural enrichment, Carbon source, Aeration, Respiration

## Abstract

**Background:**

Vitamin K2 (menaquinone, MK-n) is a lipid-soluble vitamin that functions as a carboxylase co-factor for maturation of proteins involved in many vital physiological processes in humans. Notably, long-chain vitamin K2 is produced by bacteria, including some species and strains belonging to the group of lactic acid bacteria (LAB) that play important roles in food fermentation processes. This study was performed to gain insights into the natural long-chain vitamin K2 production capacity of LAB and the factors influencing vitamin K2 production during cultivation, providing a basis for biotechnological production of vitamin K2 and in situ fortification of this vitamin in food products.

**Results:**

We observed that six selected *Lactococcus lactis* strains produced MK-5 to MK-10, with MK-8 and MK-9 as the major MK variant. Significant diversities between strains were observed in terms of specific concentrations and titres of vitamin K2. *L. lactis* ssp. *cremoris* MG1363 was selected for more detailed studies of the impact of selected carbon sources tested under different growth conditions [i.e. static fermentation (oxygen absent, heme absent); aerobic fermentation (oxygen present, heme absent) and aerobic respiration (oxygen present, heme present)] on vitamin K2 production in M17 media. Aerobic fermentation with fructose as a carbon source resulted in the highest specific concentration of vitamin K2: 3.7-fold increase compared to static fermentation with glucose, whereas aerobic respiration with trehalose resulted in the highest titre: 5.2-fold increase compared to static fermentation with glucose. When the same strain was applied to quark fermentation, we consistently observed that altered carbon source (fructose) and aerobic cultivation of the pre-culture resulted in efficient vitamin K2 fortification in the quark product.

**Conclusions:**

With this study we demonstrate that certain LAB strains can be employed for efficient production of long-chain vitamin K2. Strain selection and optimisation of growth conditions offer a viable strategy towards natural vitamin K2 enrichment of fermented foods, and to improved biotechnological vitamin K2 production processes.

**Electronic supplementary material:**

The online version of this article (10.1186/s12934-019-1179-9) contains supplementary material, which is available to authorized users.

## Introduction

Vitamin K is a fat-soluble vitamin that is essential for human health [1]. It functions as an enzyme cofactor for γ-carboxylation of glutamate (Gla) residues in Gla-proteins, which play key roles in a number of vital physiological processes including haemostasis, calcium and bone metabolism, as well as cell growth regulation [1, 2]. Vitamin K exists naturally in two forms: vitamin K1 (phylloquinone) and vitamin K2 (menaquinones). Vitamin K1 is abundantly present in green leafy vegetables and found in some vegetable oils and is the predominant form of vitamin K in our daily diet. Vitamin K2 refers to a group of menaquinones (MKs) varying in side chain length. Different forms of MKs are written as MK-n, where n indicates the number of isoprenoid residues in its side chain [3]. MK-4 is the most common form of short-chain MKs, and is produced in human and animal tissues by converting vitamin K1 or analogs of MK-precursors [3]. Meats, eggs and milk are the common dietary source of MK-4. The long-chain MKs, namely MK-5 to MK-13, are uniquely synthesized by bacteria [3]. The main dietary sources of long-chain MKs are fermented foods [3, 4].

The dietary intake of vitamin K2 covers only 10% to 25% of the total vitamin K intake in the Dutch and German population [5] and is assumed to be even lower in many other countries. The bioavailability of vitamin K2 is thought to be higher than that of vitamin K1 [6]. Compared to vitamin K1, the co-factor efficacy of vitamin K2 for protein carboxylation has been shown to be higher [7]. Notably, some forms of vitamin K2 with long side chains, e.g. MK-7 and MK-9, have been found to have longer plasma half-life times than vitamin K1 and MK-4, suggesting advantages for their uptake and utilization by the human body [7–9]. Moreover, indications have been found that dietary intake of vitamin K2, especially in the form of MK-7, MK-8 and MK-9, is associated with a reduced risk of coronary heart disease [6, 10, 11]. Vitamin K2 intake is also involved in normal bone growth and development, whereas its deficiency is associated with increased risk of fracture and low bone mineral density [2, 5, 12, 13]. Some intestinal bacteria also produce vitamin K2, but in animal studies the absorption of this vitamin in the colon was found to be limited [14] and hence dietary intake is an essential source to obtain this vitamin. This information, combined with advances in nutrition research, underline the importance of fortifying food products with vitamin K2, especially the long-chain forms, in food products and supplements.

Production of long-chain vitamin K2 has been observed in a variety of bacteria involved in well-known food fermentation processes, and the specific structure of menaquinones produced by these bacteria has been determined [3, 15]. These food grade bacteria can be seen as potential candidates for in situ fortification in fermented foods and biotechnological production of long-chain MKs. *Bacillus subtilis* produces MK-7 and some strains are used to make fermented soybean food products, among which the Japanese natto is well-known [3]. However, fermented food products involving *B. subtilis* are mostly appreciated in certain regions in Asia and do not contribute to the western diet. Several studies have been performed to optimize biotechnological production of MK-7 in *B. subtilis* [16–18]. Propionibacteria, producing MK-9 (4H), are used to produce Swiss-type cheeses [19, 20] and are applied in biotechnological MK-9 (4H) production processes [21]. Lactic acid bacteria (LAB) are key players in various food fermentation processes as starter cultures, probiotics and producers of vitamins. Among LAB, *Lactococcus lactis* ssp. *cremoris*, *L. lactis* ssp. *lactis*, *Leuconostoc lactis* and *Leuconostoc mesenteroides* are the reported producers of mainly MK-8, MK-9 and MK-10 [22]. In spite of the wide applications of LAB, studies were mainly conducted to reveal vitamin K2 levels in fermented dairy products [4, 19] and only a few studies [22, 23] collected information on vitamin K2 production in LAB in laboratory conditions. Therefore, long-chain vitamin K2 production in LAB definitely deserves more investigation.

Vitamin K2 is present in the cytoplasmic membranes of producing bacteria, acting as an electron carrier in the respiratory electron transport chain (ETC) [24]. Although LAB have been classified as non-respiring, facultative anaerobes, conclusive evidence has been found for functional respiration in various lactococci, lactobacilli, and pediococci in response to heme (for some species, menaquinone and heme) supplementation [23]. Menaquinones, together with the NADH dehydrogenase complex and the *bd*-type cytochrome complex (where heme functions as an essential cofactor), form a simple electron transport chain that enables aerobic respiration in these bacteria when oxygen is present. Nevertheless, it was found that menaquinone is produced in *L. lactis* continuously, under conditions including static fermentation (no oxygen or heme present), aerobic fermentation (oxygen present, no heme) and aerobic respiration (both oxygen and heme present) [23]. The role of menaquinones in the fermentative metabolism of producing bacteria remains unclear so far.

The vitamin K2 biosynthesis pathway begins with chorismate [25]. Chorismate is generated from shikimate pathway, that is supplied with precursors derived from glycolysis and the pentose phosphate pathway [26, 27], and thus linking the production of menaquinone to the central carbon metabolism. Therefore, the type of carbon source as well as the mode of energy metabolism potentially affects vitamin K2 synthesis in bacteria.

In this study, we focus on long-chain MKs as they are uniquely produced by bacteria and are thought to deliver additional health benefits in comparison to other vitamin K forms [6–9]. We aim to provide insights into the natural long-chain vitamin K2 producing capacity in different LAB strains and the influences of cultivation conditions on vitamin K2 production. To this end, we first examined the MK forms and quantity produced by different *L. lactis* and *Lc. mesenteroides* strains. Next, we selected *L. lactis* ssp. *cremoris* MG1363 to investigate changes in MK profile and levels in response to various growth conditions (i.e. different growth temperatures, degrees of aeration, types of carbon sources and modes of energy metabolism), and used selected parameters for vitamin K2 enrichment in a quark product.

## Materials and methods

### Bacterial strains and culture conditions

For strain screening, we examined *L. lactis* ssp. *lactis* strains FM03, FM04, DSM20481, *L. lactis* ssp. *cremoris* strains MG1363, YL11, YL12 and *Lc. mesenteroides* strains DSM20343, FM06 and FM08. *L. lactis* strains were cultivated in M17 media (Oxoid LOT2216165) supplemented with 0.5% (w/v) glucose (GM17), and *Lc. mesenteroides* strains were cultivated in MRS (De Man, Rogosa and Sharpe) media. The inoculum of each biological replicate was a single colony of the corresponding strain. Fifty-millilitre centrifuge tubes were filled with 50 ml media and statically incubated at 30 °C for 48 h.

*L. lactis* ssp. *cremoris* MG1363 was used to examine the effect of cultivation conditions. A single colony was inoculated into GM17 media and incubated at 30 °C overnight. Ten microliter of the overnight culture was inoculated in 50 ml M17 media supplemented with 0.5% (w/v) carbon source. The carbon source was glucose unless specified otherwise. For static fermentation, 50 ml cultures were incubated statically in 50 ml centrifuge tubes. For aerobic fermentation, 50 ml cultures were placed in 500 ml Erlenmeyer flasks and shaken at one of the following shaking speeds: 60 rpm, 120 rpm and 200 rpm. For aerobic respiration, 50 ml cultures supplemented with 2 µg/ml hemin (heme) (Sigma) were shaken at 120 rpm in 500 ml Erlenmeyer flasks. Bacteria were cultivated for 48 h at 30 °C, unless the temperature was otherwise stated.

### Quark fermentation

Pre-cultures for quark fermentation were obtained by inoculating *L. lactis* ssp. *cremoris* MG1363 in M17 media supplemented with 0.5% (w/v) carbon source and incubated overnight at 30 °C, with aeration (aerobic fermentation at 120 rpm) or without aeration (static fermentation). The quark making procedure is modified from Binda and Ouwehand [28]. Together with 45 ml pasteurised skim milk (Jumbo Biologische houdbare Magere Melk, Netherlands), 0.5% (w/v) carbon source, 1% (w/v) casein tryptone (Sigma) and 1% (v/v) pre-culture were added to 50 ml centrifuge tubes. Fermentation took place at 20 °C or 30 °C for 20 h, and the pH values of samples were checked to be similar to traditional quark products (4.6). After centrifugation at 3000×*g* for 10 min, the whey was discarded to obtain quark products.

### Vitamin K2 extraction

For extraction from bacterial biomass, bacteria were harvested by centrifugation at 8000×*g* for 15 min. The supernatant was discarded. The cell pellet was washed twice with 1× volume of phosphate-buffered saline (PBS). The pellet was then re-suspended in 1.5 ml PBS that contained 10 mg/ml lysozyme (Sigma) and incubated at 37 °C for 1 h. The cell suspension was vigorously mixed on a vortex at the beginning and after 30 min of incubation. An extraction buffer was made with *n*-hexane and 2-peopanol at ratio 2:1 (v/v). Four volumes of extraction buffer was added to the lysozyme-treated bacterial suspension and the mixture was vigorously vortexed for 30 s. The mixture was then centrifuged at 3000×*g* for 10 min, and the (upper) organic phase was collected. Equal volume (as extraction buffer) of *n*-hexane was added to the remaining lower phase, and the extraction step was repeated twice.

For vitamin K2 extraction from quark samples, the method was adapted from Manoury et al. [29]. Four grams of quark sample was mixed with 10 ml MQ water and vigorously vortexed for 30 s. Then 5 ml of 1 M HCl was added to the mixture, and the sample was heated at 99 °C for 30 min in a water bath and cooled in tap water. Ten millilitres iso-propanol and 4 ml hexane were added to the sample. The mixture was vigorously vortexed for 30 s and centrifuged at 3000×*g* for 10 min. The organic phase was collected. The extraction was repeated once with 4 ml hexane.

After extraction from bacteria or quark, organic phases from each sample were combined and evaporated under N_2_ gas. Iso-propanol was then added to the sample vials to dissolve the extracted vitamin K2 by shaking at 160 rpm for 1 h. All samples were diluted in methanol and subjected to high performance liquid chromatography–mass spectrometry (HPLC–MS) analysis.

### Determination of vitamin K2 content

Vitamin K2 extraction samples were analysed on a HPLC (UFLC, Shimadzu, Japan) system coupled with a Micromass Quattro Ultima MS (Waters, USA). Fifty-microliter of each sample was injected on a Symmetry C18, 5 µm, 150 × 3 mm column (Waters, USA). Elution of compounds followed a gradient that started with 100% methanol (solvent A), 0% 2-propanol/hexane 50/50 (solvent B), and changed to 25% solvent A and 75% solvent B in 10 min. Then the gradient changed to 1% solvent A and 99% solvent B in 2 min and remained for another 2 min. The flow rate was 0.4 ml/min and oven temperature was 40 °C. Eluted compounds were detected by the MS system with an atmospheric pressure chemical ionization (APCI) source in the positive mode. The corona current was 5 µA. The APCI source temperature was 120 °C and probe temperature 500 °C. Details of MRM (multiple reaction monitoring) are presented in Additional file 1: Table S1. Standards of MK-1 (Santa Cruz Biotechnology), MK-4 (Sigma), MK-7 (Sigma), MK-9 (Santa Cruz Biotechnology) and vitamin K1 (Sigma) in the concentration range from1 ng/ml to 3 µg/ml were analysed to obtain the calibration curves. Except for the strain screening experiment, vitamin K1 was added at a concentration of 300 ng/ml as an internal standard in each sample.

### Quantification of biomass

Biomass accumulation was quantified by cell dry weight (CDW) determination. The cells were harvested and washed twice in PBS as described in the vitamin K2 extraction protocol. Water content in the cell pellets was evaporated at 80 °C for 48–72 h. The dried biomass was then weighed.

### Determination of growth rate

Overnight cultures of bacteria in GM17 media, incubated statically at 30 °C were diluted in fresh GM17 to an optical density (OD) value of 0.2 measured at 600 nm. The diluted cultures were incubated statically at specific temperatures. The OD_600nm_ were measured every 30 min for in total 6 h. Growth rates were calculated by plotting the natural logarithm values of the OD_600nm_ values; slopes of the linear range from each plot were used to determine the specific growth rates.

### Data analysis

HPLC-MS data was processed in MassLynx software (Waters). Quantities of MK-5 and MK-6 were estimated using the formulas derived from MK-4 and MK-7 calibration curves, respectively; quantities of MK-8 and MK10 were estimated using the formula derived from MK-9 calibration curve. Where applicable, the peak areas in samples were corrected based on the measurement of vitamin K1 internal standards.

Vitamin K2 levels were presented in specific concentrations and titres. Specific concentrations were obtained by calculating total vitamin K2 (MK-5 to MK-10) content per unit biomass (nmol/g CDW) while titres were obtained by calculating total vitamin K2 content per volume medium that was used to harvest the biomass (nmol/L medium). In fold change calculations the control group was in all cases *L. lactis* MG1363 cultivated in GM17 media, statically at 30 °C for 48 h.

Statistical significance analysis was performed in IBM SPSS Statistics (version 23) using one-way or two-way analysis of variance (ANOVA). Original values of measurement were used for ANOVA. Homogeneity of variance was examined using Levene’s test (α = 0.05). Unless stated otherwise, the post hoc multiple comparisons were conducted using Dunnett test (2-sided) and in all cases the control group was *L. lactis* MG1363 cultivated in GM17 media, statically at 30 °C for 48 h (*P ≤ 0.05; **P ≤ 0.01; ***P ≤ 0.001).

## Results

### Strain diversity in vitamin K2 production

**Fig. 1:**
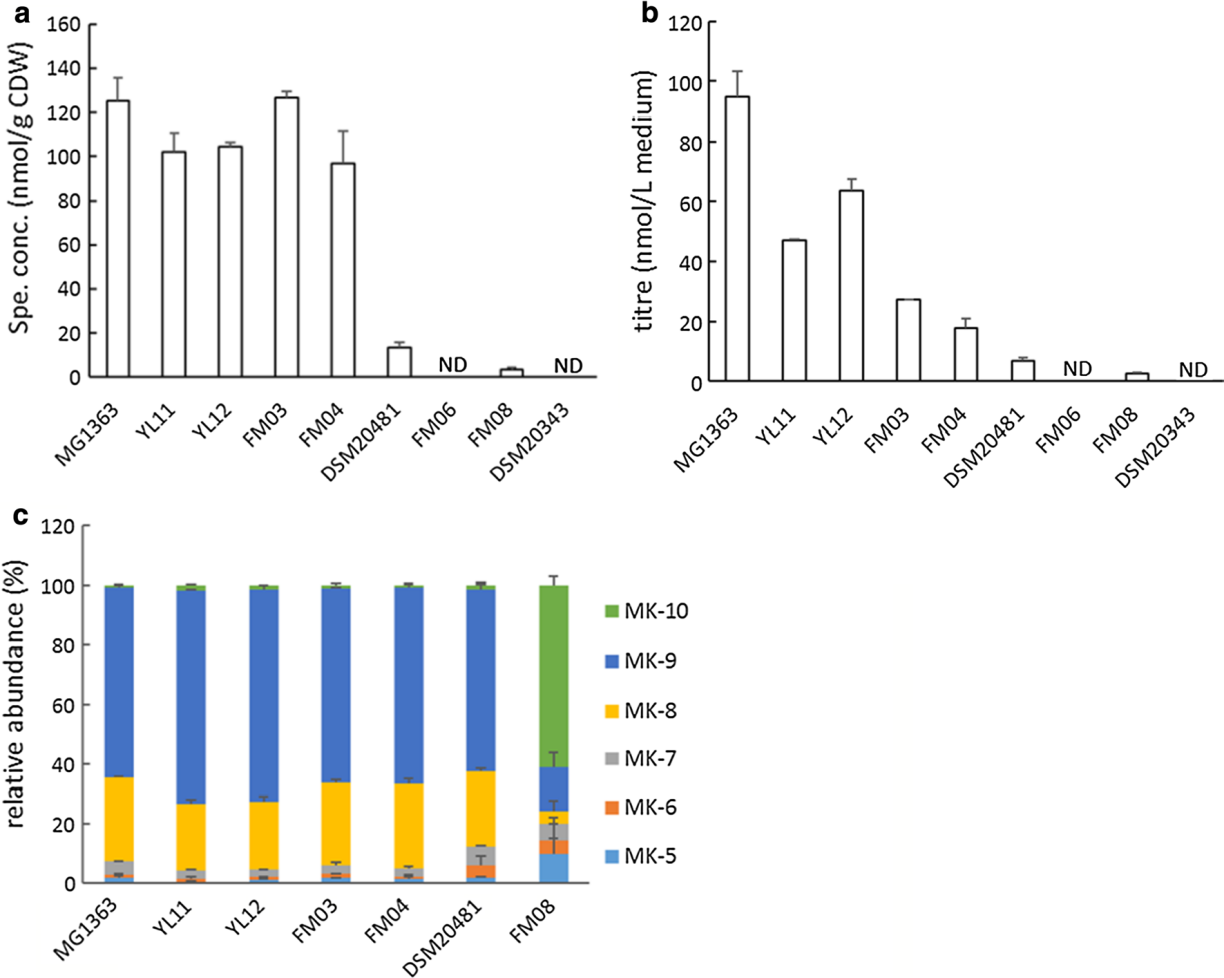
Vitamin K2 production in different LAB strains. **a** Specific concentrations of total vitamin K2. **b** Titres of total vitamin K2. **c** Relative abundance of MKs. MG1363, YL11 and YL12 are *L. lactis* ssp. *cremoris* strains; FM03, FM04 and DSM20481 are *L. lactis* ssp. *lactis* strains; FM06, FM08 and DSM20343 are *Lc. mesenteroides* strains. *L. lactis* strains were cultivated in GM17 and *Lc. mesenteroides* in MRS media, all statically incubated at 30 °C for 48 h. Values shown are from averages of biological triplicates for MG1363 and duplicates for the other strains. Error bars represent standard errors of the means (SEMs)

To reveal the natural capacity of vitamin K2 production in different LAB strains, we examined MK forms and levels in strains of *L. lactis* ssp. *lactis, L. lactis* ssp. *cremoris* and *Lc. mesenteroides* (Fig. 1). Strains of *L. lactis* ssp. *lactis* and *L. lactis* ssp. *cremoris* produced vitamin K2 in the form of MK-5 to MK-10 (Fig. 1c). MK-9 was the most abundant followed by MK-8. Among the *L. lactis* strains, a large diversity of vitamin K2 producing capacity was demonstrated by the specific concentrations: *L. lactis* ssp. *cremoris* MG1363 and *L. lactis* ssp. *lactis* FM03 produced the highest amount of vitamin K2, reaching 125 nmol/g CDW; *L. lactis* ssp. *cremoris* DSM20481 produced the lowest amount, 13 nmol/g CDW (Fig. 1a). The strain diversity was also evident as reflected by the titres (Fig. 1b). *L. lactis* ssp. *cremoris* MG1363 showed the highest titre of about 95 nmol/L medium while *L. lactis* ssp. *lactis* DSM20481 showed the lowest titre of 7 nmol/L medium. *Lc. mesenteroides* strains FM06 and DSM20343 did not produce detectable amounts of vitamin K2. *Lc. mesenteroides* FM08 produced a very low amount: about 3 nmol/g CDW in specific concentration and 2.5 nmol/L medium in titre (Fig. 1a, b). The MK composition produced in *Lc. mesenteroides* FM08 was also different from *L. lactis*: MK-10 was the major form followed by MK-9, and MK-5 to MK-8 in low abundance (Fig. 1c). Since *L. lactis* ssp. *cremoris* MG1363 produced the highest specific concentrations and titres of vitamin K2 in the tested conditions, this strain was selected for more detailed studies.

### Effect of temperature on vitamin K2 production

**Fig. 2:**
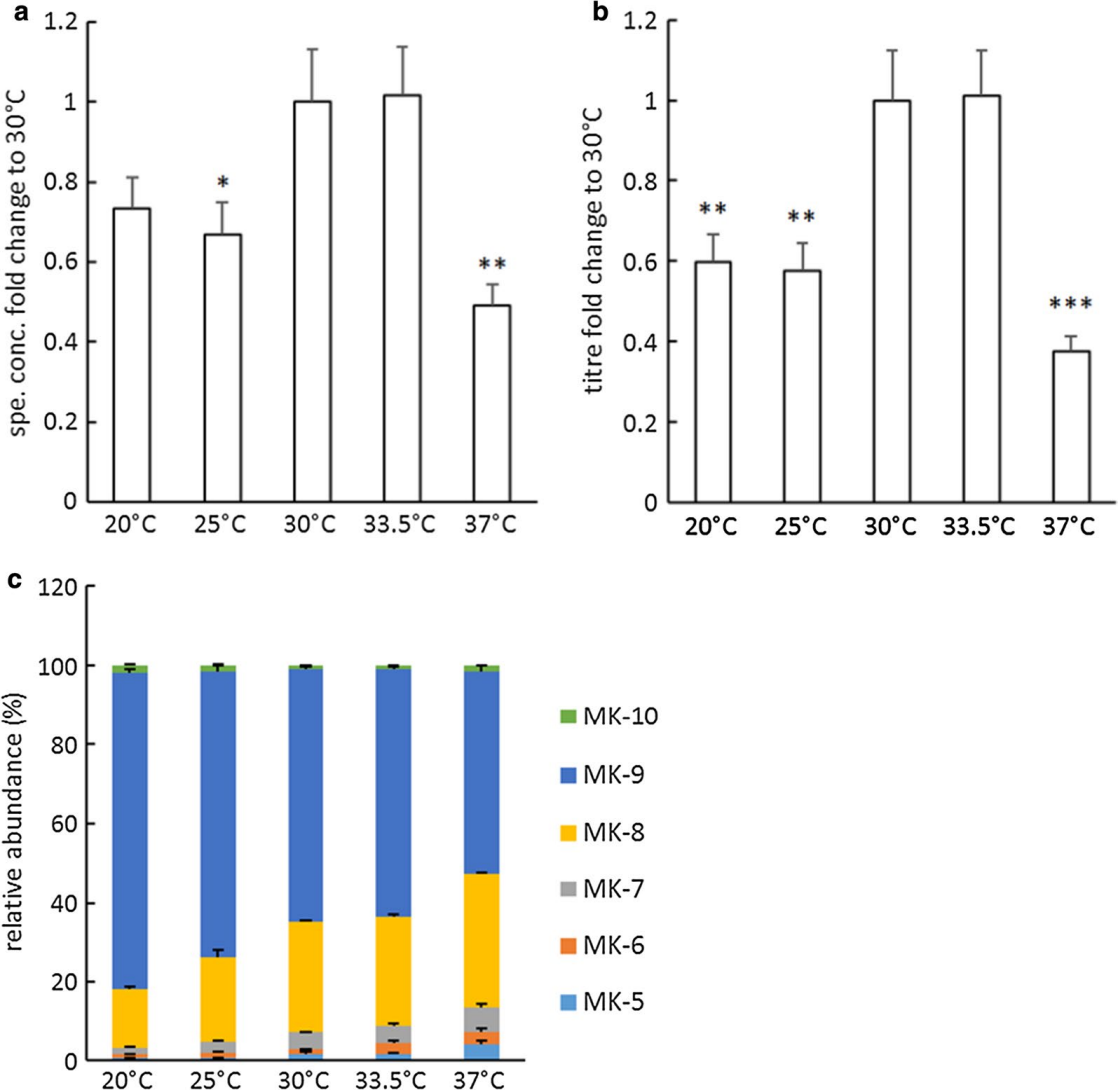
Effect of temperature on vitamin K2 production in *L. lactis* ssp. *cremoris* MG1363. **a** Fold changes in specific concentrations with respect to the control group (30 °C). **b** Fold changes in titres with respect to the control group (30 °C). **c** Relative abundance of MKs. Strain MG1363 was cultivated in GM17, statically incubated for 48 h at indicated temperature. Values shown are from averages ± SEMs of biological triplicates. For Dunnett test (2-sided) the control group was 30 °C (*P ≤ 0.05; **P ≤ 0.01; ***P ≤ 0.001)

*Lactococcus lactis* ssp. *cremoris* MG1363 was cultivated at different temperatures and the MK levels and forms were measured (Fig. 2). Significant differences were observed for specific concentrations (P = 0.001) and titres (P < 0.0005) as a result of varied temperatures. At 30 °C and 33.5 °C, the specific concentrations and titres were the highest, about two-times higher than the lowest values obtained at 37 °C (Fig. 2a, b). The relative abundance of different MK-n forms also changed under altered temperatures (Fig. 2c), and from 20 to 37 °C, the relative abundance of MK-9 gradually decreased from 80 to 50%, while other shorter-chained forms, i.e. MK-5 to MK-8, increased.

### Impact of carbon sources on vitamin K2 production

**Fig. 3:**
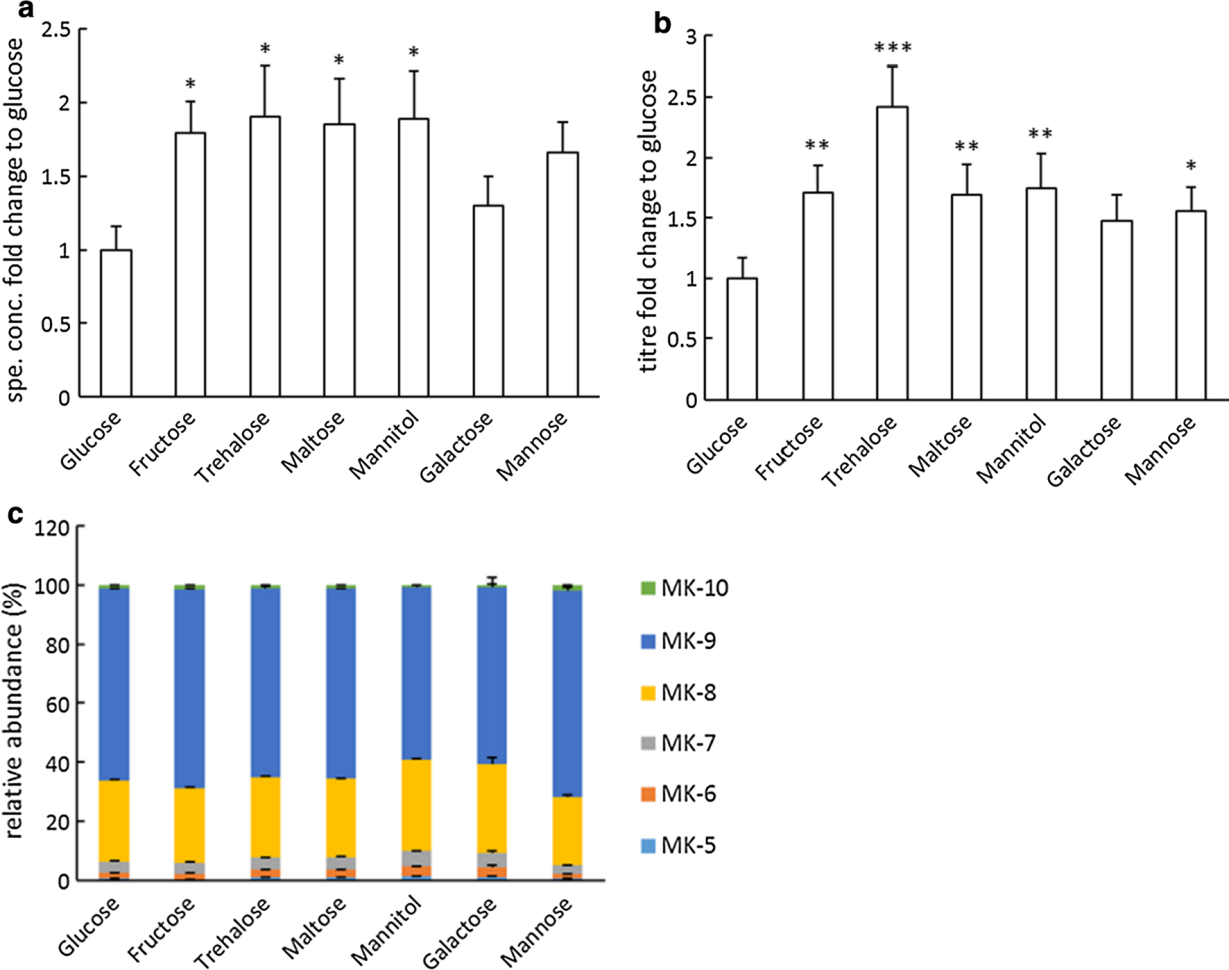
Effect of carbon source on vitamin K2 production in *L. lactis* ssp. *cremoris* MG1363. **a** Fold changes in specific concentrations with respect to the control group (glucose). **b** Fold changes in titres with respect to the control group (glucose). **c** Relative abundance of MKs. Strain MG1363 was cultivated in M17 supplemented with indicated carbon source, statically incubated at 30 °C for 48 h. Values shown are from averages ± SEMs of biological triplicates. For Dunnett test (2-sided) the control group was glucose (*P ≤ 0.05; **P ≤ 0.01; ***P ≤ 0.001)

To examine the effect of carbon source on vitamin K2 production, *L. lactis* ssp. *cremoris* MG1363 was cultivated in M17 supplemented with different carbon sources and the MK levels and forms were measured (Fig. 3). Significant differences in specific concentrations and titres of vitamin K2 production were observed (P = 0.021 and P < 0.0005 respectively) as a result of the varied carbon source supplementation. Fructose, trehalose, maltose and mannitol all resulted in an approximately 1.8-fold increase in specific concentrations compared to glucose (Fig. 3a). A similar increase in titres was also observed for the above mentioned carbon sources, with the exception of trehalose which resulted in a 2.4-fold increase compared to glucose (Fig. 3b). The relative abundance of different MKs was slightly influenced by the carbon source (Fig. 3c). Mannitol and galactose resulted in the lowest relative abundance of MK-9, ~ 60%, while mannose resulted in the highest MK-9 abundance, ~ 70%. Glucose, fructose, trehalose and maltose resulted in similar MK compositions, with ~ 65% MK-9.

### Aerobic cultivation leads to elevated vitamin K2 levels

**Fig. 4:**
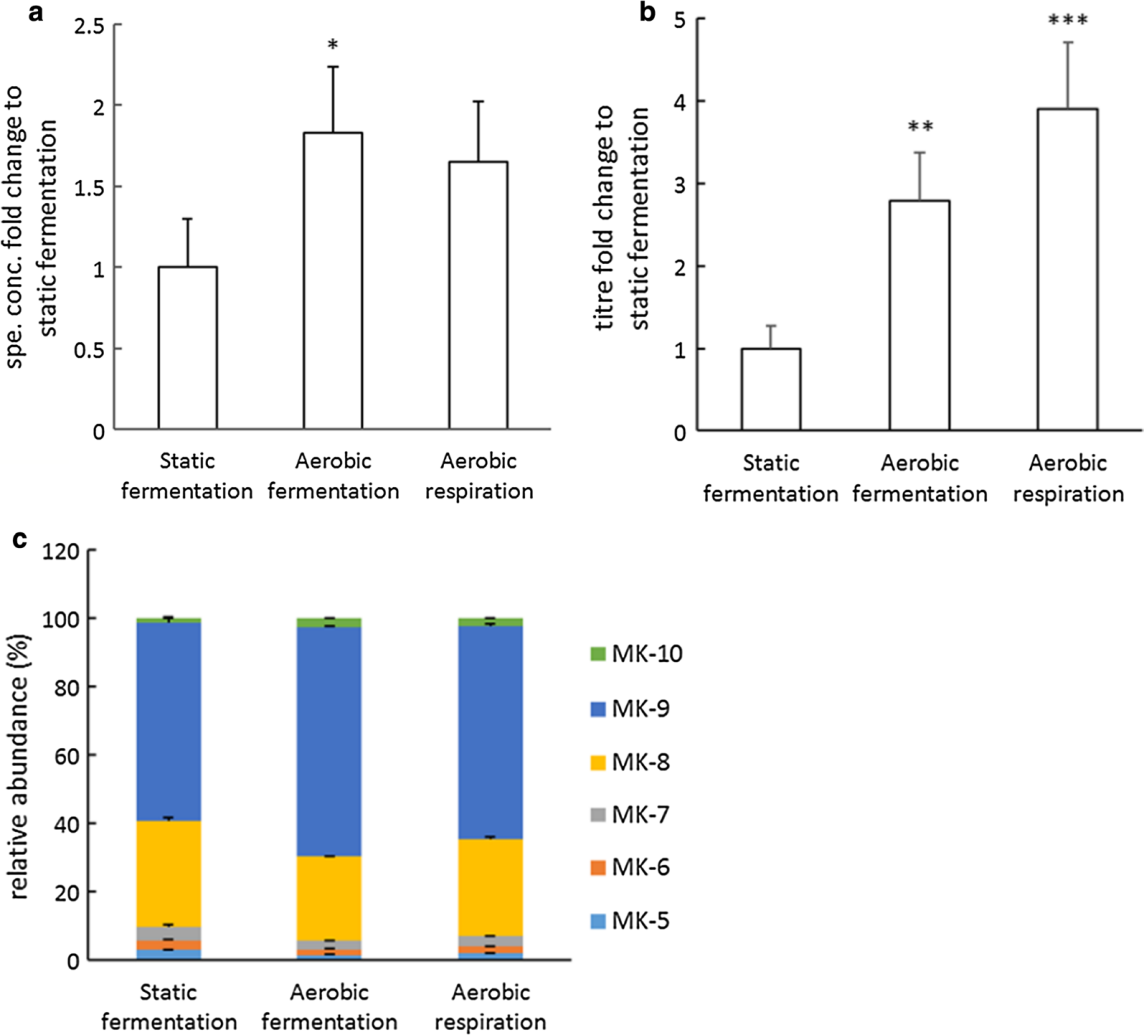
Effect of aeration and respiration on vitamin K2 production in *L. lactis* ssp. *cremoris* MG1363. **a** Fold changes in specific concentrations with respect to the control group (static fermentation). **b** Fold changes in titres with respect to the control group (static fermentation). **c** Relative abundance of MKs. Strain MG1363 was cultivated in GM17 at 30 °C for 48 h. For static fermentation, full tubes of cultures were statically incubated. For aerated fermentation, bacterial cultures were incubated in flasks with head space, shaken at 120 rpm. For respiration, bacterial cultures were incubated in flasks with head space, shaken at 120 rpm and with 2 µg/ml heme added. Values shown are from averages ± SEMs of biological triplicates. For Dunnett test (2-sided) the control group was static fermentation (*P ≤ 0.05; **P ≤ 0.01; ***P ≤ 0.001)

To examine the effect of aeration and respiratory metabolism on vitamin K2 production, *L. lactis* ssp. *cremoris* MG1363 was cultivated under conditions of static fermentation, aerobic fermentation and aerobic respiration. Specific concentrations as well as titres differed significantly among these three conditions (P = 0.034 and P < 0.0005 respectively). Aerobic fermentation resulted in a 1.9-fold increase in specific concentration, and a 2.8-fold increase in titre compared to static fermentation (Fig. 4a, b). Aerobic respiration resulted in a fourfold increase in titre compared to static fermentation. Aerobic fermentation resulted in a shift towards longer-chained MKs (25% MK-8, 67% MK-9 and 3% MK-10) in comparison to static fermentation (31% MK-8, 58% MK-9 and 1% MK-10) (Fig. 4c), and similar MK compositions were found with aerobic respiration.

**Fig. 5:**
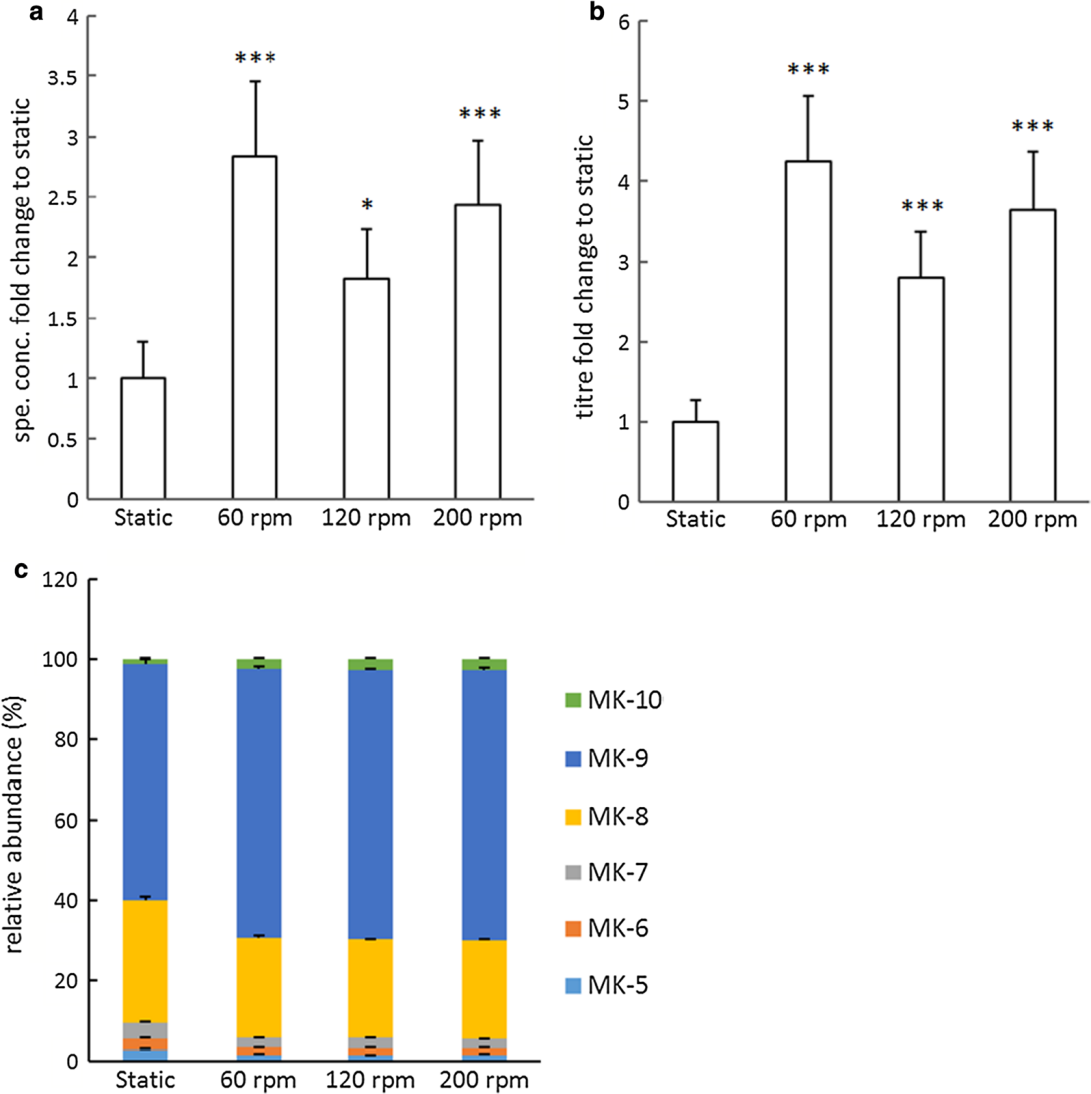
Effect of degree of aeration on vitamin K2 production in *L. lactis* ssp. *cremoris* MG1363. **a** Fold changes in specific concentrations with respect to the control group (static fermentation). **b** Fold changes in titres with respect to the control group (static fermentation). **c** Relative abundance of MKs. Strain MG1363 was cultivated in GM17 at 30 °C for 48 h. For static fermentation, full tubes of cultures were statically incubated. For aerated fermentation, bacterial cultures were incubated in flasks with head space, shaken at indicated speeds. Values shown are from averages ± SEMs of biological triplicates. For Dunnett test (2-sided) the control group was static (*P ≤ 0.05; **P ≤ 0.01; ***P ≤ 0.001)

The effect of degree of aeration on vitamin K2 production was further examined by cultivating bacteria at different shaking speeds. Aerobic fermentation at all three tested shaking speeds resulted in increased vitamin K2 specific concentrations and titres (Fig. 5a, b). The highest amount was obtained at 60 rpm, 2.8-fold increase in specific concentration and 4.3-fold increase in titre compared to static fermentation, followed by 200 rpm and 120 rpm. The values obtained at 60 rpm were also significantly higher compared to aeration at 120 rpm (Tukey HSD P = 0.037 and P = 0.006), but not compared to 200 rpm (Tukey HSD P = 0.595 and P = 0.322). The relative abundances of MKs were identical in aerated conditions with all three tested shaking speeds, all resulted in a shift towards longer-chained MKs compared to static fermentation (Fig. 5c).

### Effect of aerobic cultivation on vitamin K2 production is carbon source-dependent

**Fig. 6:**
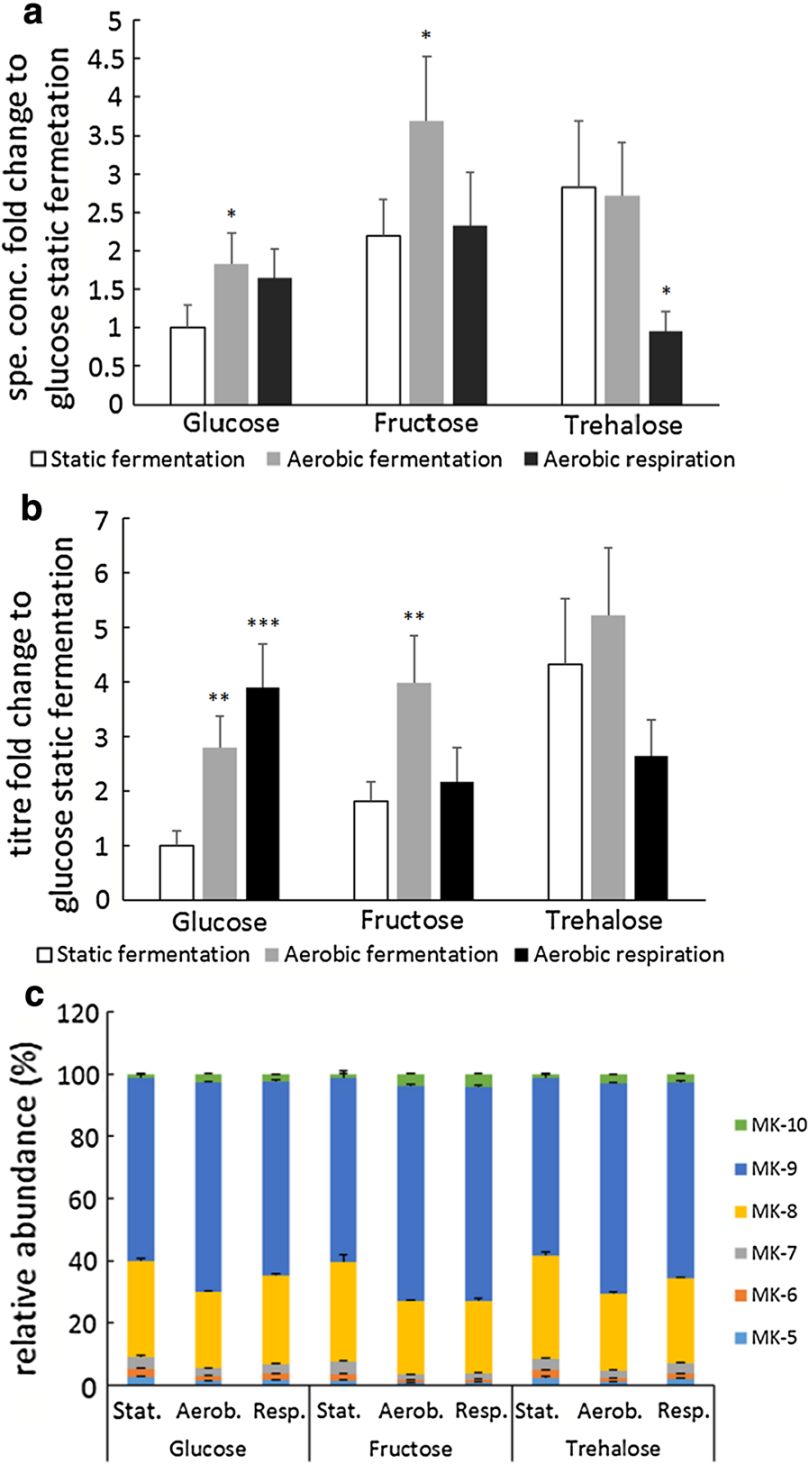
Effect of carbon source and aeration/respiration on vitamin K2 production in *L. lactis* MG1363. **a** Fold changes in specific concentrations with respect to glucose, static fermentation. **b** Fold changes in titres with respect to glucose, static fermentation. **c** Relative abundance of MKs. Strain MG1363 was cultivated in M17 with indicated carbon source at 30 °C for 48 h. For static fermentation, full tubes of cultures were statically incubated. For aerated fermentation, bacterial cultures were incubated in flasks with head space, shaken at 120 rpm. For respiration, bacterial cultures were incubated in flasks with head space, shaken at 120 rpm and with 2 µg/ml heme added. Values shown are from averages ± SEMs of biological triplicates. Dunnett test (2-sided) was performed within each carbon source group, and the control groups were in all cases static fermentation (*P ≤ 0.05; **P ≤ 0.01; ***P ≤ 0.001)

Observing that aerobic cultivations (aerobic fermentation and aerobic respiration) lead to increased vitamin K2 production when glucose was the carbon source, we further examined whether the same effect remained when other carbon sources such as fructose and trehalose were used. The effects of carbon source and aerobic cultivation were analysed by two-way ANOVA. A significant main effect of carbon source was observed (P < 0.0005 for specific concentration, P = 0.001 for titre), and so was a significant main effect of aerobic cultivation (P = 0.002 for specific concentration, P = 0.001 for titre). Moreover, a significant interaction between carbon source and aerobic cultivation was found [F(4, 21) = 4.804, P = 0.007 for specific concentration; F(4, 21) = 7.719, P = 0.001 for titre]. When fructose was the carbon source, the specific concentration and titre were both highest with aerobic fermentation while aerobic respiration resulted in similar level as static fermentation; when trehalose was the carbon source, aerobic respiration resulted in lower vitamin K2 production than static and aerobic fermentation (Fig. 6a, b). Comparing all combinations, aerobic fermentation with fructose resulted in the highest specific concentration, i.e. a 3.7-fold increase compared to static fermentation with glucose. Aerobic fermentation with trehalose resulted in the highest titre, 5.2-fold compared to the same control group. The change in relative abundance of MKs in the three carbon sources across different conditions were similar: static fermentation resulted in the lowest abundance of MK-9, MK-10 and highest abundance of MK-8; a shift towards longer-chained MKs was observed in aerobic respiration and further in aerobic fermentation (Fig. 6c).

### Vitamin K2 fortification in quark

Quark products were made from pasteurised milk using *L. lactis* ssp. *cremoris* MG1363 as the starter culture. We varied carbon sources and aeration conditions for making the pre-cultures, as well as the carbon sources and temperatures in quark fermentation. All quark samples showed pH values around 4.9. All quark samples were fortified with vitamin K2 compared to unfermented milk (1.4 ± 0.1 ng/g), with MK-7 to MK-10 being the detectable forms (Table 1). Fermentation at 30 °C (B, 72.1 ± 12.9 ng/g) resulted in a 61% increase of total vitamin K2 content in the quark samples compared to fermentation at 20 °C (A, 44.9 ± 10.6 ng/g). Under the same pre-culturing condition, using fructose as a carbon source for fermentation, we found an 18% to 73% increase in the vitamin K2 content in quark samples compared to using glucose as the carbon source (B vs. C, D vs. E and F vs. G). Moreover, the use of aerated pre-cultures resulted in 35% to 79% increased vitamin K2 content in quark samples compared to static pre-cultures (D vs. F, E vs. G).

**Table 1:** Vitamin K2 content in quark samples

No.^a^	Pre-culture		Quark		MK-7	MK-8	MK-9	MK-10	Total VK2
	C source^b^	Aeration	C source	Temp.^c^ (°C)	(ng/g of quark; means ± SEMs^d^)				
A	Glucose	No	Glucose	20	0.8 ± 0.3	5.4 ± 0.8	36.7 ± 10.0	0.4 ± 0.1	44.9 ± 10.6
B	Glucose	No	Glucose	30	1.0 ± 0.3	16.0 ± 5.8	52.1 ± 8.6	1.5 ± 1.2	72.1 ± 12.9
C	Glucose	No	Fructose	30	1.0 ± 0.3	16.2 ± 2.2	64.8 ± 8.2	1.0 ± 0.5	85.0 ± 10.3
D	Fructose	No	Glucose	30	0.7 ± 0.2	7.5 ± 1.5	36.3 ± 10.4	1.8 ± 1.1	48.8 ± 13.0
E	Fructose	No	Fructose	30	2.0 ± 0.5	18.8 ± 5.2	59.9 ± 9.6	0.9 ± 0.4	84.4 ± 15.6
F	Fructose	Yes	Glucose	30	1.9 ± 0.3	15.9 ± 3.3	64.9 ± 5.8	1.4 ± 0.1	87.3 ± 9.1
G	Fructose	Yes	Fructose	30	1.7 ± 0.4	18.9 ± 4.8	88.1 ± 8.8	2.6 ± 1.1	113.8 ± 9.8

^a^Each combination of cultivation condition was given a number for easy reference in the text

^b^Carbon source

^c^Temperature

^d^All conditions was tested in biological triplicates, except for B which was in duplicate

## Discussion

Long-chain vitamin K2 production in LAB is of interest for food industry, but there has not been much effort made to optimize conditions for vitamin K2 production and to elucidate the influencing environmental factors. In this study we examined the levels and forms of MKs (MK-5 to MK-10) from different LAB strains, and the influence of cultivation conditions. In our study, the levels of total vitamin K2 production are expressed as specific concentrations (in nmol vitamin K2 per gram cell dry weight). The values of specific concentrations show the natural vitamin K2 producing capacity of bacteria and may provide insights into the bacterial physiology related to the synthesis of these membrane-embedded menaquinones. Titres were derived from the amount of vitamin K2 recovered from the biomass harvested from the culture medium, expressed as nmol vitamin K2/L medium, and therefore showed a combined effect of vitamin K2 accumulation in the cells and biomass accumulation in the media. The values of titres provide clear information on the product output for a certain amount of medium/material input, and therefore is of strong industrial interest.

We screened *L. lactis* ssp. *cremoris*, *L. lactis* ssp. *lactis* and *Lc. mesenteroides* strains, as these LAB (sub)species are known to possess the complete set of genetic elements for menaquinone synthesis [30] and are frequently used in food fermentation processes. From each (sub)species, the prototype (MG1363) or type strain (DSM20481, DSM20343), together with two other strains isolated from fermented foods, were selected to show a representative picture of the diversity among vitamin K2-producing LAB species and strains. Comparative analysis of vitamin K2 production showed that the specific concentrations and titres of the six selected *L. lactis* strains varied largely, demonstrating a wide strain diversity in the natural vitamin K2 producing capacity (Fig. 1a, b). The specific concentration values were in the same order of magnitude as reported previously for *L. lactis* [22]. It is also clear that specific concentrations and titres may not show the same trend. As vitamin K2 is cell membrane-associated, the titres are not only determined by specific concentrations but also biomass yields. For example, strain FM03 showed one of the highest specific concentrations but its titre value was among the lowest due to its low biomass accumulation in the culture media (Additional file 1: Figure S1). We detected no significant amounts of vitamin K2 in *Lc. mesenteroides* FM06 and DSM20343 and only very minor amounts in FM08 in the tested conditions. Although a complete set of menaquinone-synthesis genes have been identified in *Lc. mesenteroides*, Brooijmans et al. [23] also observed the discrepancy that they could not induce respiratory growth in the tested *Lc. mesenteroides* strain.

Analysis of the relative abundance of long-chain MKs in *L. lactis* strains showed very similar distributions, and MK-8 and MK-9 were found to be the most abundant (Fig. 1c). The minor amount of vitamin K2 produced by *Lc. mesenteroides* strain FM08 was found to contain MK-10 as the major form. Given the large strain diversity, strain selection can be an efficient first step towards improved vitamin K2 fortification in fermented foods or food supplements. Both high vitamin K2 producing capacity and high biomass accumulation under selected cultivation conditions should be criteria for strain selection to achieve vitamin K2 fortification. For this reason, we selected prototype strain *L. lactis* MG1363, which showed both high specific concentration and high titre, to further analyse the impact of selected cultivation parameters on vitamin K2 production in *L. lactis*.

Using *L. lactis* MG1363 as the model strain, we demonstrated that vitamin K2 production is influenced by temperature, carbon source, aeration and mode of energy metabolism. Temperature influenced both the total amount and relative abundance of different long-chain MK forms in MG1363. At 30 °C to 33.5 °C the specific concentrations and titres were the highest (Fig. 2a, b), temperatures above or below this range resulted in lower vitamin K2 production. As the temperature increased from 20 to 37 °C, MK-8 became more abundant and MK-9 less (Fig. 2c). This could be caused by the influence of temperature on the activity of the enzyme that controls the isoprenoid side chain elongation during menaquinone synthesis. As MKs are membrane embedded, their relative abundance could also be influenced by the dynamics in biomass accumulation, including the growth rate (shown in Additional file 1: Table S2), composition of lipids and fatty acid side chains, and membrane fluidity, which are all affected by temperature.

The impact of different carbon sources on vitamin K2 production was also significant. Fructose, trehalose, maltose and mannitol all resulted in about twofold increase of vitamin K2 production compared to glucose (Fig. 3a, b). Trehalose stood out for the highest titre level, as it also lead to the highest biomass accumulation (Additional file 1: Figure S3). This is one of the first studies to examine the effect of using trehalose for metabolite production in LAB. Our study demonstrates a positive effect of various carbon sources compared to glucose, on vitamin K2 production in *L. lactis*.

Vitamin K2 is known for its function as electron carrier in the bacterial ETC, and *L. lactis* can switch to respiratory metabolism when both oxygen and heme are supplied [23]. Moreover, the presence of oxygen alone has impact on metabolism by altering the balance of redox cofactors, as well as expression and activity of key enzymes [31]. Therefore, it was highly relevant to examine whether vitamin K2 production was influenced by aeration only (fermentation under aerobic conditions) and respiratory metabolism (aerobic respiration). We demonstrated production of MK-5 to MK-10 under static fermentation, aerobic fermentation and aerobic respiration conditions, and a shift towards longer chained MKs under the two aerobic conditions (Fig. 4). This is in agreement with the study from Brooijmans et al. [23] regarding the quantity of MK-5 to MK-10. We observed increased specific concentrations of MK-5 to MK-10 under both aerobic conditions compared to static fermentation, which could suggest specific roles of long-chain MKs in aerobic conditions. Moreover, as the increase was observed for both aerobic fermentation and aerobic respiration, it is likely that MK production responded to oxygen, and not necessarily a functional respiratory ETC. A transcriptomic analysis from Cretenet et al. [32] also revealed that the first gene in menaquinone-synthesis pathway, *menF*, is upregulated by 2.7-fold during the early stage of cultivation under oxygen condition compared to anaerobic condition in MG1363. Pedersen et al. [33] also detected up-regulation of another menaquinone-synthesis enzyme, *menB*, under aerobic condition compared to static cultivation in a *L. lactis* strain. Increased activity of the menaquinone-synthesis pathway could explain the increased vitamin K2 content in aerobically cultivated *L. lactis* MG1363 cells. The increase of MK production was even more obvious in terms of titres, since besides higher specific concentrations, the biomass accumulations in aerobic fermentation and aerobic respiration were also higher than in static fermentation (Additional file 1: Figure S4). A higher biomass yield under aerobic conditions was also reported by Nordkvist et al. and they propose that this was because of less energy limitation for biomass synthesis [31]. Respiratory metabolism is known to enhance the growth efficiency in *L. lactis* [23], and indeed we obtained the highest biomass accumulation with aerobic respiration, reaching a more than twofold increase compared to static fermentation.

When we further examined the aerobic fermentation conditions by applying different degrees of aeration, we observed that all aerobic fermentation conditions clearly resulted in enhanced vitamin K2 production compared to static fermentation (Fig. 5a, b), and the enhancement did not seem to be a function of the degree of aeration.

When the effects of aeration and respiratory metabolism were examined with carbon sources other than glucose, differences in response were observed (Fig. 6a, b). With fructose as the carbon source, aerobic fermentation resulted in the highest vitamin K2 production, while aerobic respiration was similar to static fermentation. When trehalose was used, aerobic fermentation resulted in similar level as static fermentation, while aerobic respiration resulted in much lower vitamin K2 production. Therefore, the effect of aerobic fermentation and aerobic respiration on vitamin K2 production was found to be carbon source-dependent. It was found to be consistent though, for all 3 carbon sources tested, the biomass accumulation was highest under aerobic respiration, followed by aerobic fermentation, and lowest under static fermentation (Additional file 1: Figure S6). The MK profile also reflected that for all 3 carbon sources, aerated conditions lead to a shift towards longer chained MKs compared to static fermentation (Fig. 6c).

The reason why each carbon source or cultivation condition influenced vitamin K2 production in this way remains to be elaborated. As a secondary metabolite, the metabolic fluxes towards vitamin K2 are complicated with a variety of enzymatic reactions involved. The flux towards chorismate, which is the first compound in menaquinone biosynthesis pathway, could be influenced by the central carbon metabolism. As different carbon sources are taken up, converted and directed to the central carbon metabolism, the conversion rate, energy transduction, redox factor regeneration and regulation of the sugar catabolism all vary [34, 35]. This could globally explain why the amount of vitamin K2 production in *L. lactis* responded to aeration as well as respiration in a carbon source-dependent manner. A series of genes are required for menaquinone synthesis, some dedicated to the naphthoquinone synthesis, some to the elongation of the isoprenoid tail. Together, they influence the quantity and composition of the menaquinone pool. However, to date, regulations of these genes under different conditions have been hardly investigated [36], and little is known about feedback regulation in this pathway, especially in LAB. A mechanistic explanation for the response towards each specific carbon source or cultivation condition requires further studies and the construction of a comprehensive metabolic model.

We further demonstrated that the obtained knowledge can be transferred to food fermentation processes by fortifying quark with vitamin K2. Consistently, cultivation temperature of 30 °C, altered carbon source (fructose) and aerobic cultivation of the pre-culture, resulted in higher vitamin K2 content in the quark product.

The insights obtained from this study show a proof of principle that strain selection and combination of favourable cultivation parameters, namely temperature, aeration, carbon source and mode of energy metabolism, can contribute to improved production of long-chain vitamin K2 in LAB strains. As hydrophobic, membrane-embedded compounds, menaquinones are not produced extracellularly and continuously, but remain cell-associated in all producing bacteria. Nevertheless, simple and efficient procedures are being continuously developed and optimized for extracting vitamin K2 and other valuable cell-associated molecules from biomass [37–39], that, together with the knowledge obtained from this study, will facilitate the biotechnological production of long-chain vitamin K2.

The effect of different carbon sources also demonstrate the potential of long-chain vitamin K2 fortification during fermentation of different raw food materials, enabling utilization of low-value substrates in industrial processes or development of vitamin K2-enriched fermentation-based food products as exemplified in the current study with the production of vitamin K2-fortified quark. Since vegetables like cabbage [40], beetroots and carrots [41] contain high levels of fructose, they could serve as excellent sources for in situ vitamin K2 fortification through fermentation with selected LAB.

## Conclusion

In this study, we demonstrated a large species and strain diversity in LAB for vitamin K2 producing capacity. Furthermore, the influence of relevant cultivation conditions on vitamin K2 production in *L. lactis* MG1363 has been studied in detail. We concluded that vitamin K2 production is already significantly affected when a single factor among temperature, carbon source, aeration and mode of metabolism, is changed. The highest specific concentration of vitamin K2 was achieved under aerobic fermentation with fructose, reaching 3.7-fold increase compared to static fermentation with glucose; the highest titre was found under aerobic respiration with trehalose, reaching 5.2-fold increase compared to static fermentation with glucose. The ratios of MK-8 to MK-10 increased in response to aerobic cultivations. Knowledge obtained from this study can contribute to biotechnological production of vitamin K2 supplements as well as to strategies for natural enrichment of vitamin K2 in fermented foods, and together with further studies lead to better understanding of the regulation of vitamin K2 production and its role in LAB metabolism and physiology.

## Additional file

**Additional file 1: Figure S1.** Biomass accumulation in different LAB strains. **Figure S2.** Effect of temperature on biomass accumulation in *L. lactis* ssp. *cremoris* MG1363. **Figure S3.** Effect of carbon source on biomass accumulation in *L. lactis* ssp. *cremoris* MG1363. **Figure S4.** Effect of aeration and respiration on biomass accumulation in *L. lactis* ssp. *cremoris* MG1363. **Figure S5.** Effect of degree of aeration on biomass accumulation in *L. lactis* ssp. *cremoris* MG1363. **Figure S6.** Effect of different carbon source and aeration/respiration on biomass accumulation in *L. lactis* ssp. *cremoris* MG1363. **Table S1.** Details of MRM analysis. **Table S2.** Specific growth rate of *L. lactis* ssp. *cremoris* MG1363 at different temperatures.

## Data Availability

All data generated or analysed during this study are included in this published article and its additional files.

## Abbreviations

MK: menaquinone
LAB: lactic acid bacteria
ETC: electron transport chain
HPLC–MS: high performance liquid chromatography–mass spectrometry
CDW: cell dry weight
OD: optical density
ANOVA: analysis of variance
PBS: phosphate-buffered saline
