# Supplementary material for: Long-chain vitamin K2 production in Lactococcus lactis is influenced by temperature, carbon source, aeration and mode of energy metabolism
Source: Microb Cell Fact. 2019 Aug 6;18:129. doi: 10.1186/s12934-019-1179-9 (PMC6683496; doi:10.1186/s12934-019-1179-9)
Supplement: Supplementary file 1 — Additional file 1: Figure S1. Biomass accumulation in different LAB strains. Figure S2. Effect of temperature on biomass accumulation in L. lactis ssp. cremoris MG1363. Figure S3. Effect of carbon source on biomass accumulation in L. lactis ssp. cremoris MG1363. Figure S4. Effect of aeration and respiration on biomass accumulation in L. lactis ssp. cremoris MG1363. Figure S5. Effect of degree of aeration on biomass accumulation in L. lactis ssp. cremoris MG1363. Figure S6. Effect of different carbon source and aeration/respiration on biomass accumulation in L. lactis ssp. cremoris MG1363. Table S1. Details of MRM analysis. Table S2. Specific growth rate of L. lactis ssp. cremoris MG1363 at different temperatures. [file 12934_2019_1179_MOESM1_ESM.docx]

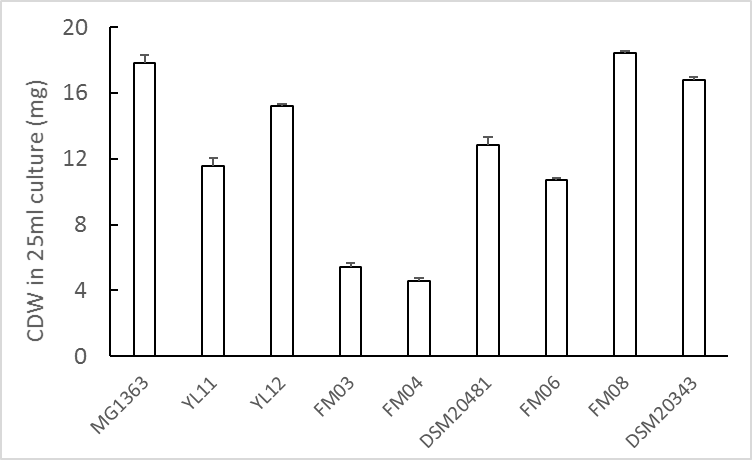


Figure S1. Biomass accumulation in different LAB strains. *L. lactis* strains were cultivated in GM17 and *Leu. mesenteroides* in MRS media, all statically incubated at 30°C for 48 h. Values shown are from averages of biological triplicates for MG1363 and duplicates for the other strains. Error bars represent standard errors (SEs).


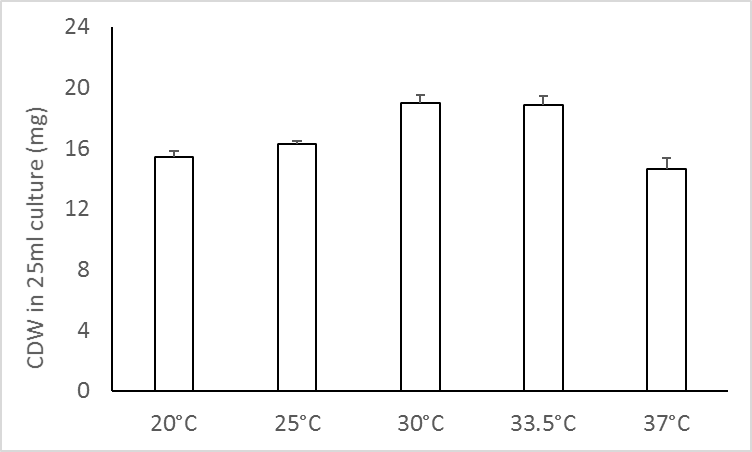


Figure S2. Effect of temperature on biomass accumulation in *L. lactis* ssp. *cremoris* MG1363. Strain MG1363 was cultivated in GM17, statically incubated for 48 h at indicated temperature. Values shown are from averages ± SEs of biological triplicates.


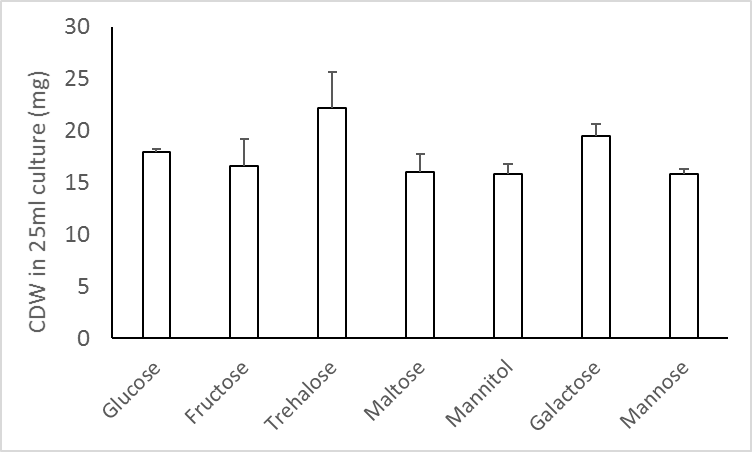


Figure S3. Effect of carbon source on biomass accumulation in *L. lactis* ssp. *cremoris* MG1363. Strain MG1363 was cultivated in M17 supplemented with indicated carbon source, statically incubated at 30°C for 48 h. Values shown are from averages ± SEs of biological triplicates.


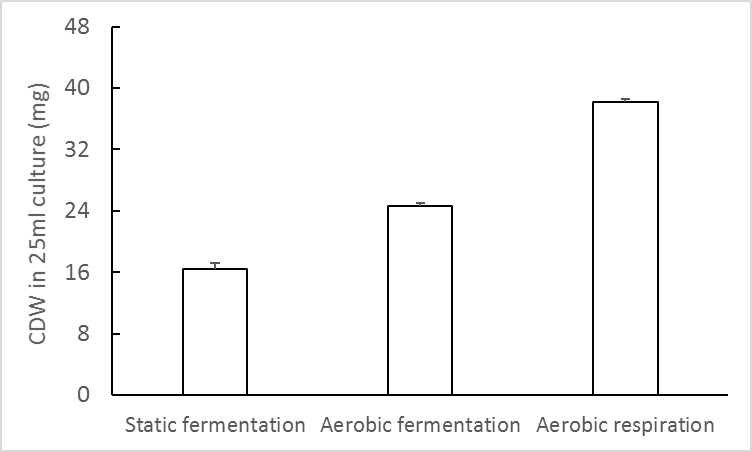


Figure S4. Effect of aeration and respiration on biomass accumulation in *L. lactis* ssp. *cremoris* MG1363. Strain MG1363 was cultivated in GM17 at 30°C for 48 h. For static fermentation, full tube of culture was statically incubated. For aerated fermentation, bacterial culture was incubated in flasks with head space, shaken at 120 rpm. For respiration, bacterial culture was incubated in flasks with head space, shaken at 120 rpm and with 2 µg/ml hemin added. Values shown are from averages ± SEs of biological triplicates.


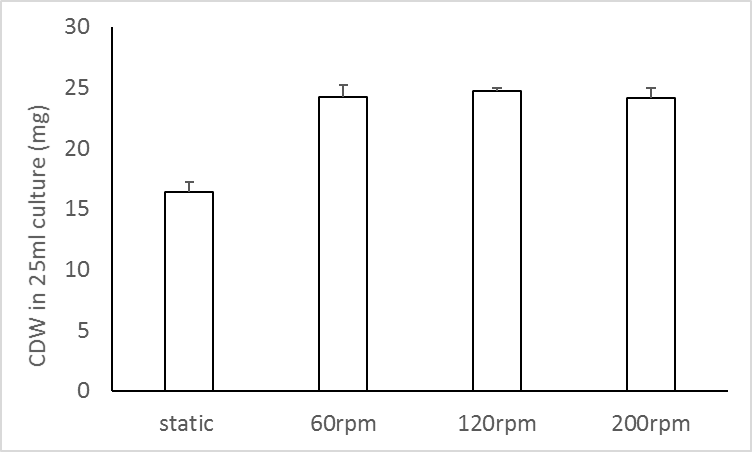


Figure S5. Effect of degree of aeration on biomass accumulation in *L. lactis* ssp. *cremoris* MG1363. Strain MG1363 was cultivated in GM17 at 30°C for 48 h. For static fermentation, full tube of culture was statically incubated. For aerated fermentation, bacterial culture was incubated in flasks with head space, shaken at indicated speeds. Values shown are from averages ± SEs of biological triplicates.


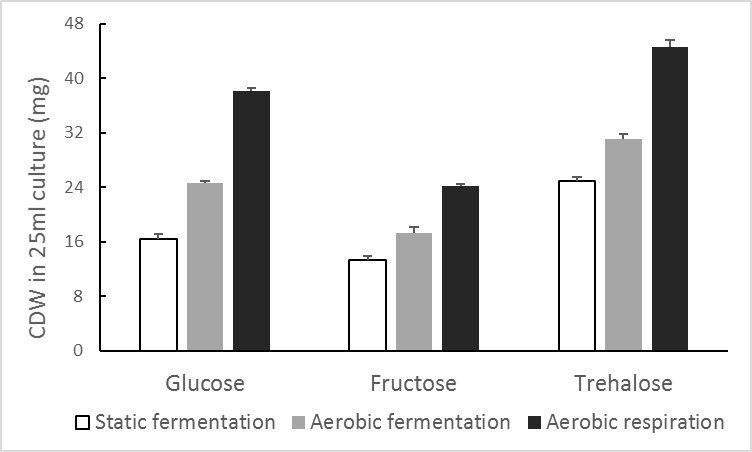


Figure S6. Effect of different carbon source and aeration/respiration on biomass accumulation in *L. lactis* ssp. *cremoris* MG1363. Strain MG1363 was cultivated in M17 with indicated carbon source at 30°C for 48 h. For static fermentation, full tube of culture was statically incubated. For aerated fermentation, bacterial culture was incubated in flasks with head space, shaken at 120 rpm. For respiration, bacterial culture was incubated in flasks with head space, shaken at 120 rpm and with 2 µg/ml hemin added. Values shown are from averages ± SEs of biological triplicates.

Table S1. Details of MRM analysis. Different forms of MKs were detected in 3 functions. Dwell time was in all cases 0.08 s in function 1 and 2, and 0.10 s in function 3. Cone voltage was 30.00 V in all cases.

| Function | Molecule | Parent ion (Da) | Daughter ion (Da) | Collision energy (eV) |
| --- | --- | --- | --- | --- |
| 2 | Vitamin K1 | 451.55 | 187.25 | 25.00 |
| 2 | Vitamin K1 | 451.55 | 227.28 | 20.00 |
| 2 | MK-5 | 513.37 | 187.25 | 25.00 |
| 2 | MK-5 | 513.37 | 227.28 | 20.00 |
| 2 | MK-6 | 581.44 | 187.25 | 25.00 |
| 2 | MK-6 | 581.44 | 227.28 | 20.00 |
| 2 | MK-7 | 649.76 | 187.25 | 25.00 |
| 2 | MK-7 | 649.76 | 227.28 | 20.00 |
| 2 | MK-8 | 717.56 | 187.25 | 25.00 |
| 2 | MK-8 | 717.56 | 227.28 | 20.00 |
| 3 | MK-9 | 785.72 | 187.25 | 35.00 |
| 3 | MK-9 | 785.72 | 227.28 | 30.00 |
| 3 | MK-10 | 853.69 | 187.25 | 35.00 |
| 3 | MK-10 | 853.69 | 227.28 | 30.00 |

Table S2. Specific growth rate of *L. lactis* ssp. *cremoris* MG1363 at different temperatures. Strain MG1363 was cultivated in GM17 at 30°C with a starting OD=0.2. Growth was followed by OD measurement for 5 hours, and maximum growth rates were determined for the exponential phase of each culture.

| Growth temperature (°C) | Growth rate (h-1) |
| --- | --- |
| 20 | 0.54 |
| 25 | 0.72 |
| 30 | 0.87 |
| 37 | 1.07 |
